# A Double-Blind, Randomized Clinical Trial of Niacinamide 4% versus Hydroquinone 4% in the Treatment of Melasma

**DOI:** 10.1155/2011/379173

**Published:** 2011-07-21

**Authors:** Josefina Navarrete-Solís, Juan Pablo Castanedo-Cázares, Bertha Torres-Álvarez, Cuauhtemoc Oros-Ovalle, Cornelia Fuentes-Ahumada, Francisco Javier González, Juan David Martínez-Ramírez, Benjamin Moncada

**Affiliations:** ^1^Department of Dermatology, Hospital Central, Universidad Autónoma de San Luis Potosí, San Luis Potosí, Mexico; ^2^Department of Dermatology, Hospital Central “Dr. Ignacio Morones Prieto”, 2395 Venustiano Carranza Avenue, CP 78210, San Luis Potosí, SLP, Mexico; ^3^Department of Pathology, Hospital Central, Universidad Autónoma de San Luis Potosí, San Luis Potosí, SLP, Mexico; ^4^Coordinación para la Innovación y la Aplicación de la Ciencia y la Tecnología, Universidad Autónoma de San Luis Potosí, San Luis Potosí, SLP, Mexico

## Abstract

*Background*. Multiple modalities have been used in the treatment of melasma with variable success. Niacinamide has anti-inflammatory properties and is able to decrease the transfer of melanosomes. *Objective*. To evaluate the therapeutic effect of topical niacinamide versus hydroquinone (HQ) in melasma patients. *Patients and Methods*. Twenty-seven melasma patients were randomized to receive for eight weeks 4% niacinamide cream on one side of the face, and 4% HQ cream on the other. Sunscreen was applied along the observation period. They were assessed by noninvasive techniques for the evaluation of skin color, as well as subjective scales and histological sections initially and after the treatment with niacinamide. *Results*. All patients showed pigment improvement with both treatments. Colorimetric measures did not show statistical differences between both sides. However, good to excellent improvement was observed with niacinamide in 44% of patients, compared to 55% with HQ. Niacinamide reduced importantly the mast cell infiltrate and showed improvement of solar elastosis in melasma skin. Side effects were present in 18% with niacinamide versus 29% with HQ. *Conclusion*. Niacinamide induces a decrease in pigmentation, inflammatory infiltrate, and solar elastosis. Niacinamide is a safe and effective therapeutic agent for this condition.

## 1. Introduction

Melasma is defined as an acquired chronic hypermelanosis on sun exposed areas being most frequently found in women with III-V phototypes of Fitzpatrick. The etiology is not completely elucidated; however, the ultraviolet sunlight exposure appears to be the most significant factor [1]. The basis of the treatment is photoprotection. Diverse modalities in drug therapy have been used such as hydroquinone (HQ), which inhibits the tyrosinase enzyme activity. In spite of its serious adverse effects and moderate results in 80% of patients, HQ is considered the gold standard treatment in melasma although usually relapses after suspension [2]. 

Niacinamide studies have demonstrated a suppression of melanosome transfer suggesting the reduction of cutaneous pigmentation [3], but to date there has been no clinical report of this effect in melasma. There have been several reports regarding other beneficial effects of topical niacinamide on the skin, including prevention of photoimmunosuppression and photocarcinogenesis [4], anti-inflammatory effects in acne [5], rosacea [6], and psoriasis [7]. It also increases biosynthesis of ceramides, as well as other stratum corneum lipids with enhanced epidermal permeability barrier function [8]. Moreover, its antiaging effects have been demonstrated in randomized trials [9].

The guidelines to clinical trials in melasma have suggested a correct diagnosis by using at least two subjective methods (besides an objective method), a comparison with the therapeutic gold standard and an evaluation of safety outcome [10].

The aim of this work was to assess the efficacy and safety of niacinamide 4% versus HQ 4% in the treatment of melasma through subjective and objective methods.

## 2. Patients and Methods

This is a double-blind, left-right randomized clinical trial. The protocol was reviewed and approved by the ethic committee in our hospital, and each subject signed a written informed consent. The sample size was determined based on favorable response: 0.8 for HQ and at least 0.4 for niacinamide, with 95% IC, two tails, *α* of 0.05 and *β* of 0.8.

We included 27 women with melasma attending the outpatient clinic of Dermatology Department at the Hospital Central “Dr. Ignacio Morones Prieto”, from March 2008 to February 2009.

Our inclusion criteria were women at least 18 years old without any topical, systemic, laser, and surgical treatment on face during the previous year. The exclusion criteria were pregnant and nursing women, patients with history of hypersensitivity to some of the components of the formulas of the study, and coexistence of associate diseases and other pigmentation diseases.

A history was taken from each patient, regarding age, gender, occupation, time of onset, history of pregnancy, contraceptive pills, and sun exposure.

At baseline, we obtained two 2 mm biopsies in 27 patients, one biopsy from lesioned and another one from facial not photoexposed skin; these were stained with haematoxylin and eosin to determine the general histopathological features of the epidermis and dermis. 

The inflammatory infiltrate was counted manually by two independent blinded observers, using a 0.5 × 0.5 mm ocular grid and 100× magnification. The cells were counted for the entire section, and the results expressed as the number of cells per mm^2^. The same procedure was employed to count melanocytes (Fontana Masson) and metachromatic granules (Wright-Giemsa) in mast cells. To count the epidermal melanin, we obtained a magnification of 40× to get a scanning view of the epidermis. Images were obtained from the entire 2 mm sample with a digital camera mounted on a microscope (Olympus CX 31) which was connected to a personal computer (PC). The image signals taken by the PC were evaluated using Image-Pro Plus Version 4.5 (Media Cybernetics, Silver Spring, MA, USA). With the aim of discern possible abnormalities of melanin in melasma patients as shown before [11], or even being induced by the intervention, we perform a qualitative analysis by Raman spectrophotometry (Horiba, Jobin-Yvon T64000. Edison, NJ, USA) before and at the conclusion of the study.

Patients were randomized in a double-blind manner to receive one treatment on the left and the other on the right side of the face. They received two containers labeled right or left with 4% niacinamide (Nicomide-T cream 4%, DUSA Pharmaceuticals Inc.) or 4% HQ (Eldoquin cream 4%, Valeant Pharmaceutical). All patients were instructed to apply the correct amount of both treatments and to use a SPF 50+ broad spectrum sunscreen every 3 hours during day time. 

Concomitant use of other skin care products or systemic treatments was not allowed during the study. Treatment was administered for the period of 8 weeks, with basal evaluation and followup at 4 and 8 weeks. Assessments included a skin pigment evaluation by a chromameter (CR-300; Minolta, Osaka, Japan), melasma area and severity index (MASI), physician global assessment (PGA) by an independent observer, conventional photography, and infrared thermography (Flexcam S, Infrared solutions, USA) with photographic register which mainly was used to detect irritation. All side-effects were registered. The double-blinded study was opened at 8 weeks in order to take a 2 mm biopsy in the side treated with niacinamide.

For statistical analysis, we used the Student *t*-test and *X* ^2^, and a *P* value of less than 0.05 was considered significant.

## 3. Results

Twenty-seven female patients with melasma were included, 12 (33%) were of skin phototype IV, and 13 (48%) of type V. The pattern of melasma was centrofacial in 13 (50%), malar in 10 (37%), and mandibular in 4 (14%). 

The patients age ranged from 25 to 53 years (mean, 37 years). The duration of melasma varied from 4 to 8 years (mean, 6.5 years). Family history of melasma was found in 19 (70%) patients. The most frequent precipitating factor was the sun exposure followed by pregnancy. Eight patients (29%) have used oral contraceptives.

### 3.1. Clinical Results

**Figure 1:**
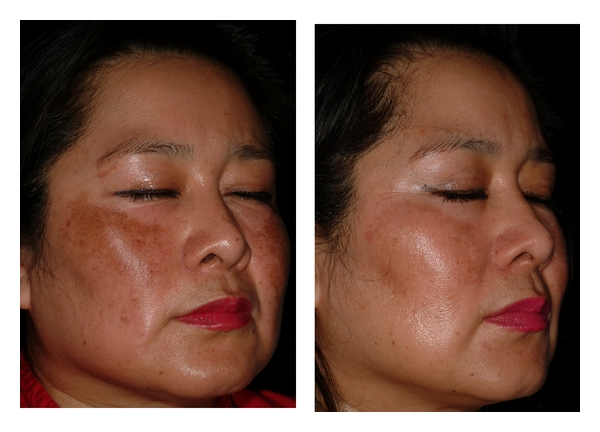
Right side treated with niacinamide. View at onset and 8 weeks later with an excellent decrease in pigmentation.

**Figure 2:**
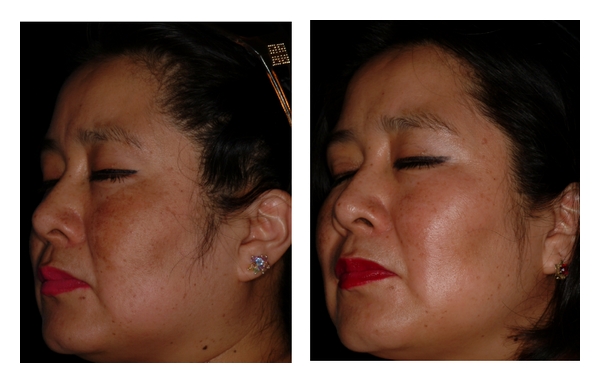
Left side treated with HQ: Onset and 8 weeks later with an excellent improvement.

The onset average MASI score for the HQ side was 4 (5% CI, 90.9–1.8) and 1.2 (95% IC, 0.8–1.6) after eight weeks (*P* < 0.001). The initial MASI score for the niacinamide side was 3.7 (95% CI, 2.9–4.4) and 1.4 (95% CI, 3.3–4.7) at the end of the study (*P* < 0.001). The average decrease for HQ was 70% and 62% for niacinamide. This improvement was registered using conventional photography (Figures 1 and 2) with no perceptible differences between both sides. 

**Figure 3:**
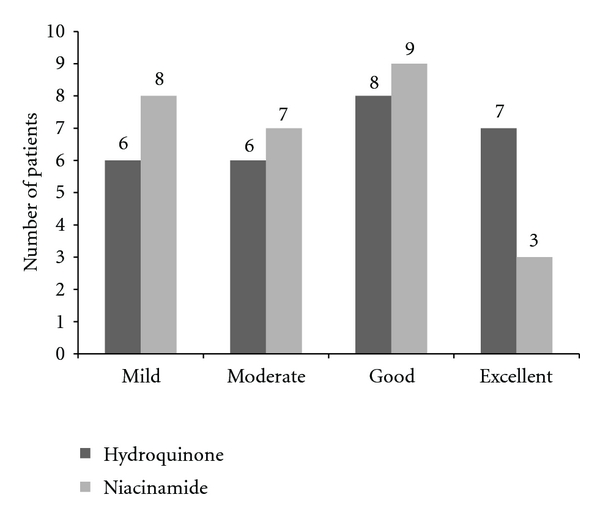
Physicians Global Assessment in melasma patients with niacinamide versus HQ.

The PGA rated the niacinamide side improvement as excellent in three patients, good in nine, moderate in seven, and mild in eight. The HQ-treated side was rated excellent in seven, good in eight, moderate in six, and mild in six patients (Figure 3). Data showed statistical significance for both treatments, HQ (*P* = 0.003), and niacinamide (*P* = 0.005).

Colorimetric assessment was performed initially and at the end of the study; we evaluated the luminosity axis (L*) as well as the erythema axis (a*). The lightening effect of HQ and niacinamide was apparent at 4 weeks of treatment, whereas it was more evident at 8 weeks. Colorimetric measures showed no statistical differences between both treatments (Table 1). The erythema was more intense on the side treated with HQ than with niacinamide, but it was not statistically significative. Infrared light thermography at environmental temperature of 21°C showed a diminished temperature of 0.8°C in both sides after treatment. There was no statistical difference between both treatments.

### 3.2. Histopathology Results

The biopsy samples were stained with haematoxylin and eosin for general histology, Fontana Masson to evaluate melanin pigment, and Wright-Giemsa for metachromatic granules in mast cells. At baseline, we found a moderate to severe degree of rete ridge flattening and epidermal thinning in 23 (85%) melasma biopsies. Solar elastosis was present in all melasma samples. Mild to moderate perivascular lymphohistiocytic infiltrates were also present in all of them and moderate presence of mast cells near elastotic areas in 11 (40%) patients. With Fontana-Masson stained sections, the amount of melanin was increased in all epidermal layers of melasma skin; we observed pigment basal cells protruded into the dermis in 20 (74%) biopsies as informed before [12]. In the upper dermis, we found scattered melanin in 19 (70%) melasma biopsies. The features in the biopsies of nonexposed sun skin were close to normal skin.

**Figure 4:**
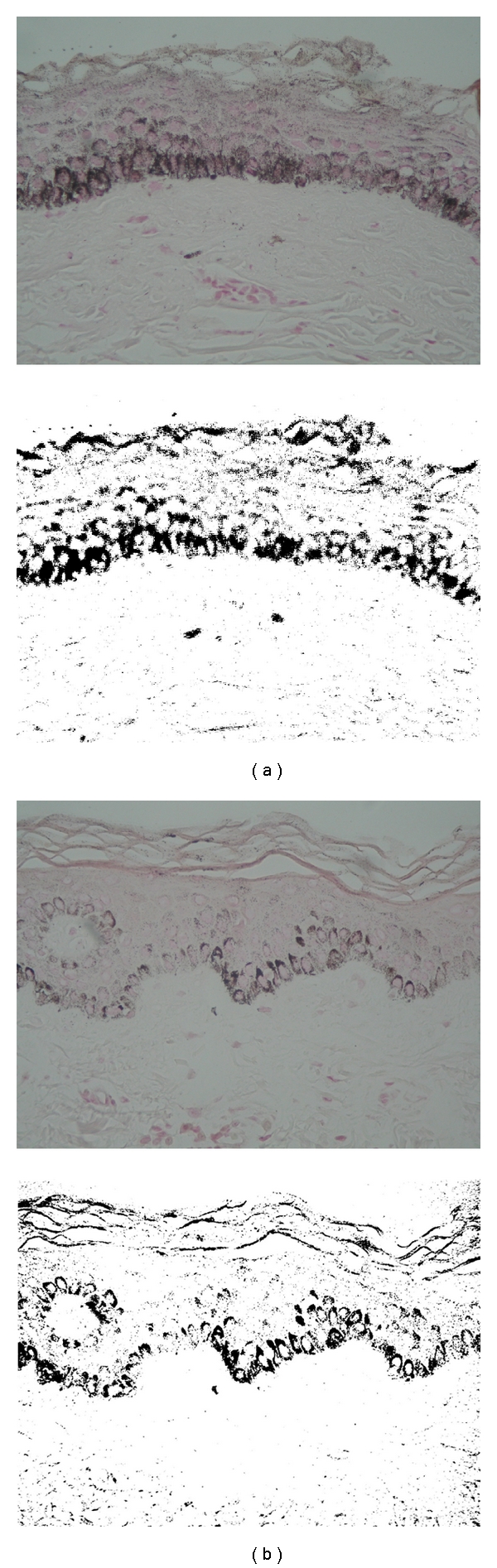
Epidermal pigmentation reduction. (a) Basal melasma skin biopsy, (b) skin biopsy posttreated with niacinamide. (Fontana Masson, original magnification 40x). Below is shown the measured positive areas for melanin using a computer-assisted image analysis program.

After 8 weeks of treatment, the blind was opened in order to take a biopsy from the side treated with niacinamide. We obtained 11 posttreatment biopsies for analysis. By means of digital analysis of biopsies images, we could observe that the amount of epidermal stained melanin was diminished significantly (*P* < 0.0007). The average inflammatory infiltrate of mast cells was reduced from 22 to 16 cells/mm^2^ (*P* = 0.01). Solar elastosis was also reduced, but no statistical differences were present (Figure 4).

### 3.3. Spectrophotometry

Raman spectroscopy measurements showed that the molecular structure of melanin was normal and remained unaltered after exposure to niacinamide since the measurements showed the characteristic peaks of melanin previously published [11, 12]. Patients with abnormal melanin could respond differently to treatment and explain the variable success rate to HQ [11]. We wanted to show that these patients were homogeneous in this aspect.

### 3.4. Side Effects

Side effects were present in the niacinamide side in 5 patients (18%), compared to 8 patients (29%) for the HQ side. The most frequent side effects were erythema, pruritus, and burning. Most of them were mild for niacinamide and moderated for HQ. On the niacinamide side, erythema, pruritus, or burning was present in 2 (7%) patients, and on the HQ side they were present in 5 (18%) patients. All these were reduced through continuous treatment in both modalities, as the a* colorimetric value did not show significant changes for both treatments at the end of the study.

## 4. Discussion

Melasma is a chronic and persistent hyperpigmentation, representing a therapeutic challenge because of the high rate of relapses. This work showed that niacinamide 4% is an effective agent for the treatment of melasma, as assessed by objective methods and clinical evaluation. Our results indicate that 4% niacinamide was effective in approximate 40% of patients, showing outstanding clinical results. In the posttreatment biopsies, we could observe that the amount of epidermal melanin and inflammatory infiltrate was diminished significantly, as well as solar elastosis although it was not enough to get statistical difference. This insufficient antiaging effect could be related to the short time of the study; therefore, further clinical studies using niacinamide for longer periods are warranted in this condition. We observed that the evolution time of melasma did not affect the response to treatment. On the other hand, colorimetric assessment showed no statistical difference between these two treatments (*P* = 0.78). However, the lightening effect of HQ was evident as early as the first month of treatment, whereas with niacinamide was noted at second month. HQ had the disadvantage of moderate adverse effects in 18% of patients, compared to milder in 7% with niacinamide. Treatment with niacinamide showed no significant side effects and was well tolerated; therefore, it could be used for longer periods, as part of the initial hyperpigmentation treatment and as maintenance drug. However, further trials are required to assess the combination of this topical drug with others agents and assess its additive effects in the treatment of melasma. The mechanism of action of niacinamide in melasma could be through the reduction of melanosomes transfer [3], photoprotection actions [4], its anti-inflammatory properties [5], and a direct or related antiaging effects such as reduction of solar elastosis [9]. We previously have described prominent infiltrates of mast cells in the elastotic areas of melasma skin [1] and evidence of damage to epidermal basal membrane, which could facilitate the fall or the migration of active melanocytes and melanin into the dermis allowing the constant hyperpigmentation in melasma [13]. Due to these findings, we wanted to prove an intervention capable of inducing modifications to these atypical findings related in the pathogenesis of melasma, in addition to modify the increased pigmentation. Therefore, we propose niacinamide as an effective, integral, and safe therapeutic alternative in the melasma treatment, since it not only reduces pigmentation and inflammation, but also may reduce solar degenerative changes with minimal adverse events.

## Figures and Tables

**Table 1:** Changes in MASI scores and colorimetric values for 4% HQ and 4% niacinamide-treated sides in 27 patients with melasma. Mast cell counts and melanin expression initially and after treatment with niacinamide in 11 patients.

	HQ			Niacinamide		
	Onset	8 wk	*P*	Onset	8 wk	*P*
MASI	4 (.9–1.8)	1.2 (.8–1.6)	<0.001	3.7 (2.9–4.4)	1.4 (3.3–4.7)	<0.001
L* axis	50.9 (49.1–52.6)	56 (54.6–57.4)	<0.001	51.1(49.3–52.9)	56 (54.4–57.5)	<0.001
a* axis	12.9 (12.3–13.5)	13.6 (12.6–14)	0.25	12.8 (12.1–13.5)	13.6 (12.7–14.5)	0.08
Mast cells/mm^2^	—	—	—	22 (15.1–28.9)	16.3 (11–21.7)	0.01
Stained Melanin (%)	—	—	—	8.7 (8–9.5)	6 (5.1–6.9)	<0.001

MASI score was calculated for each treated half-face.

No statistical differences were found in redness (a*) for both treatments, before and after.

Final depigmentation Improvement (L*) did not show differences between both treatments (*P* = 0.78). In parenthesis, confidence Interval to 95%.
